# Alterations in Spontaneous Brain Activity and Functional Network Reorganization following Surgery in Children with Medically Refractory Epilepsy: A Resting-State Functional Magnetic Resonance Imaging Study

**DOI:** 10.3389/fneur.2017.00374

**Published:** 2017-08-03

**Authors:** Yongxin Li, Zhen Tan, Jianping Wang, Ya Wang, Yungen Gan, Feiqiu Wen, Qian Chen, Derek Abbott, Kelvin K. L. Wong, Wenhua Huang

**Affiliations:** ^1^Guangdong Provincial Key Laboratory of Medical Biomechanics, School of Basic Medical Sciences, Southern Medical University, Guangzhou, China; ^2^Department of Pediatric Neurosurgery, Shenzhen Children’s Hospital, Shenzhen, China; ^3^The Second Affiliated Hospital of Guangzhou Medical University, Guangzhou, China; ^4^Centre for Biomedical Engineering, School of Electrical and Electronic Engineering, University of Adelaide, Adelaide, SA, Australia; ^5^School of Medicine, Western Sydney University, Campbelltown, NSW, Australia

**Keywords:** medically refractory epilepsy, children, surgery, amplitude of low-frequency fluctuation, functional connectivity

## Abstract

For some patients with medically refractory epilepsy (MRE), surgery is a safe and effective treatment for controlling epilepsy. However, the functional consequences of such surgery on brain activity and connectivity in children remain unknown. In the present study, we carried out a longitudinal study using resting-state functional magnetic resonance imaging in 10 children with MRE before and again at a mean of 79 days after surgery, as well as in a group of 28 healthy controls. Compared with the controls, children with epilepsy exhibited abnormalities in intrinsic activity in the thalamus, putamen, pallidum, insula, hippocampus, cerebellum, and cingulate gyrus both before and after surgery. Longitudinal analyses showed that the amplitude of low frequency fluctuations (ALFF) increased in the parietal–frontal cortex and decreased in the deep nuclei from pre- to post-surgery. The percentage changes in ALFF values in the deep nuclei were positively correlated with the age of epilepsy onset. Functional connectivity (FC) analyses demonstrated a reorganization of FC architecture after surgery. These changes in brain activity and FC after surgery might indicate that the previously disrupted functional interactions were reorganized after surgery. All these results provide preliminary evidence that the age of epilepsy onset may have some potential to predict the outcome of brain functional reorganization after surgery in children with MRE.

## Introduction

Epilepsy is the second most common neurological disease worldwide. Currently, the mainstay of epilepsy management is therapy with antiepileptic drugs. However, previous studies have shown that up to one-third of all patients with epilepsy are resistant to medical treatment (1). It is now widely accepted that epilepsy surgery is a safe and effective treatment in children diagnosed with medically refractory epilepsy (MRE) (2, 3). Successful epilepsy surgery not only controls seizures, but also substantially improves patients’ cognition, behavior, and quality of life (4). Despite these behavioral observations, the exact neural mechanisms underlying these phenomena remain obscure.

Recently, the non-invasive technique of functional magnetic resonance imaging (fMRI) has played a prominent role in the investigation of the neural mechanisms underlying epilepsy in the human brain. Prior neuroimaging studies have primarily focused on adults with epilepsy and on the evaluation of patients under consideration for epilepsy surgery (2, 5). Functional neuroimaging studies have found that functional abnormalities in epileptic patients are not limited to the epileptogenic region and that region with neuronal connections to the entire brain can also be impacted (6, 7). With surgical intervention, there is a cortical reorganization of function in the language-specific cortex and the default mode network (8–10). Nevertheless, very little is known about the functional consequences of epilepsy surgery in children. Moreover, few studies have longitudinally assessed brain activity changes after neurosurgery in children with MRE and how these neural changes relate to clinical characteristics. To the best of our knowledge, only three studies have used fMRI to examine brain activity changes after neurosurgery in children with MRE (11–13). All of these longitudinal studies in children have demonstrated restoration of functional connectivity (FC) architecture, especially in the thalamo-cortical circuitry (13). However, these three publications are case studies. Therefore, the restoration of functional plasticity following surgery needs to be validated in a larger cohort to clarify the neural mechanisms underlying these changes.

The aim of the present study was to identify a pattern of intrinsic brain activity in children diagnosed with MRE. Furthermore, we used resting-state fMRI to assess the location and extent of intrinsic brain activity after surgery and to investigate the causes of these changes. The amplitude of low frequency fluctuations (ALFF) of resting-state fMRI has been widely used to investigate the aberrant intrinsic brain activity of clinical patients (14). Recent studies have validated the high temporal stability of this approach in identifying potential biomarkers for neurological diseases (15–17). Mesial temporal lobe epilepsy patients showed a decrease in ALFF in the default mode network and an increase in ALFF in the mesial temporal, thalamus, and other cortical regions. ALFF analysis may provide a useful tool in fMRI for the study of epilepsy (18). FC is a widely used metric obtained from resting-state fMRI that measures the temporal correlation of neuronal activity-induced signal variations in anatomically different brain regions (19). This technique has also been used to capture functional brain changes in response to epilepsy treatment in adults (9, 10). Before surgery, temporal lobe epilepsy patients showed abnormal neural activity in the frontal gyri and default mode network. Post-surgery data revealed that the epilepsy patients showed a recovery of the activity in these networks. Therefore, it is potentially feasible and valuable to use this technique to investigate the response to surgery in children with MRE.

In the present study, we applied these techniques to a group of children with MRE in whom resting-state fMRI data were acquired before and after surgery. The ALFF values were calculated to compare the differences in these patients to those in the matched controls. Longitudinal changes in ALFF and FC from pre- to post-surgery were identified to explore the neural mechanism of functional plasticity after surgery. We also investigated the influence of clinical factors on ALFF in children with MRE. Based on previous research in epilepsy, we hypothesized that spontaneous brain activity and the functional network of epilepsy-related areas in children with MRE would change compared with normal children. Abnormal activity and network architecture may reorganize with surgical treatment.

## Materials and Methods

### Subjects

**Figure 1:**
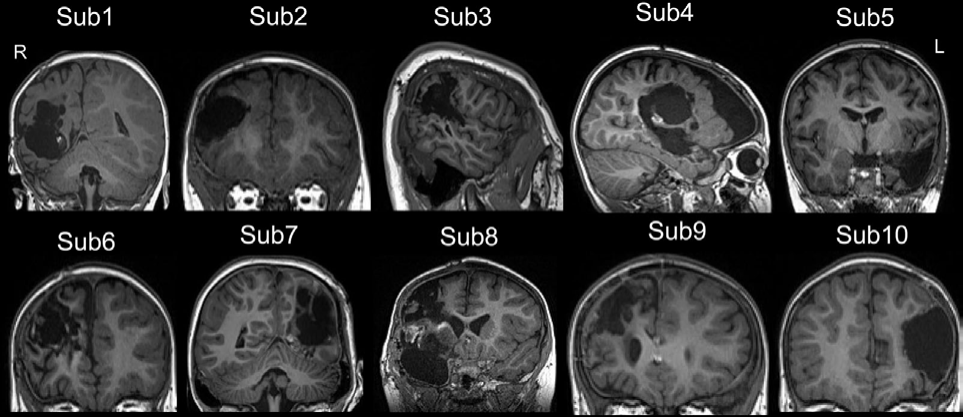
The post-surgical structural image of all patients. All images were achieved from the high-resolution T1-weighted 3D images. The images were showed in coronal or sagittal view of each subject. Surgery location: right hemisphere (Sub 1–4, Sub 6, Sub 8, and Sub 9), left hemisphere (Sub 5, Sub 7, and Sub 10). The detail of epilepsy type and the clinical information can be seen in Table 1. L, left; R, right.

Ten children with MRE [three females, age (mean ± SD): 49.94 ± 48.52 months] participated in this study. The diagnosis and location of the seizure foci were determined by a comprehensive evaluation including a detailed history and video-EEG telemetry. The details of epilepsy type and the clinical information are presented in Table 1. All patients underwent a pre-surgical evaluation and subsequently underwent resection surgery for the treatment of their epilepsy at the Shenzhen Children Hospital in Shenzhen, China. All patients who underwent epilepsy surgery were seizure free at the time of post-surgical testing. Post-surgical structural images for all patients are shown in Figure 1. Resting-state fMRI data were collected for all patients at two-time points: before and after surgery. Same imaging protocol was used for the pre- and post-surgical scans. Twenty-eight sex- and age-matched healthy controls [10 females, age (mean ± SD): 53.21 ± 37.99 months] were also recruited. These control subjects had no history of neurological disorders or psychiatric illnesses. All control subjects were scanned only once, on the day when they were recruited for this study. Written informed consent forms were obtained from all participant’s parents in accordance with the standards of the Declaration of Helsinki. The Ethics Committee of the Shenzhen Children Hospital approved this study.

**Table 1:** Summary of the clinical characteristics of child epilepsy patients.

Patient no.	Sex	Age 1 (months)	Age 2 (months)	Age 3 (months)	Interval 1 (months)	Interval 2 (months)	Pathogency	Type	Antiepileptic drugs	Seizure frequency (pre- to postoperative)
1	F	7	13	3	4	6	R cortex dysplasia	Infantile spasm	VPA, OXC, TPM	5–10/day to seizure free
2	M	9	14	0.5	9	4.5	R frontal dysplasia	Asymmetric tonic-closure seizures	VPA, CBZ, TPM	1–2/day to seizure free
3	M	161	165	36	125	4	R parietal occupation lesions	Frontal epilepsy	VPA, CBZ, TPM	2–3/day to seizure free
4	M	18	19.5	6	13	0.5	R cerebromalacia	Infantile spasm	VPA, CBZ, LEV	3–15/day to seizure free
5	F	93.5	95	12	82	1	L temporal lesion	Temporal epilepsy	OXC, VPA	3–4/day to seizure free
6	F	26.3	28	12	14.5	1.5	R frontal dysplasia	Frontal epilepsy	CBZ, VPA	2–3/day to seizure free
7	M	81	83	6	76	1	L cerebromalacia	Asymmetric tonic-closure seizures	VPA, CBZ, TPM, LEV	3–4/day to seizure free
8	M	44	48	6	38	4	R temporal lesion	Temporal epilepsy	CBZ, VPA	1/day to seizure free
9	M	27	28.5	14	13	1.5	R frontal occupation lesions	Frontal epilepsy	OXC, VPA	2–3/day to seizure free
10	M	32	35	27	6	2	L frontal occupation lesions	Asymmetric tonic-closure seizures	OXC, VPA	7–8/day to seizure free

*F, female; M, male; L, left; R, right; Age 1, age of first scan in months; Age 2, age of second scan; Age 3, age of epilepsy onset; Interval 1, interval between epilepsy onset to surgery; Interval 2, interval between surgical procedure and second scan; CBZ, carbamazepine; LEV, levetiracetam; OXC, oxcarbazepine; TPM, topiramate; VPA, valproic acid*.

### Image Acquisition

Imaging data were acquired on a 3 T Siemens scanner (MAGNETOM Trio Tim, Siemens, Germany) using an eight-channel head coil at the Shenzhen Children’s Hospital in Shenzhen, China. Foam cushions were used in the scanning procedures to reduce head translational and rotational movements. Resting blood oxygen level-dependent data were acquired from each subject using a same echo-planar imaging sequence with the following scan parameters: TR/TE = 2,000/30 ms, FOV = 220 mm × 220 mm, matrix = 94 × 94, flip angle = 90°, slice thickness = 3 mm, 36 interleaved axial slices, and 130 volumes. During the 260 s resting-state fMRI scan, all participants under the age of 4 were sedated with 10% chloral hydrate. Others were instructed to rest, to not think of anything, to keep their eyes open, and to not fall asleep. To avoid the subjects’ falling asleep, a relatively short scan was performed in this study. We also asked about their condition after the scan.

### Data Preprocessing

The resting-state fMRI data were processed using Data Processing Assistant for Resting-State fMRI software (http://www.restfmri.net). Images from the three patients with left-side epileptic pathogency (Patients no. 5, 7, 10) were oriented around the midsagittal plane prior to data analyses, thereby lateralizing the epilepsy type to the right hemisphere in all patients. The first 10 functional images per subject were excluded from analyses to ensure equilibrium of magnetization. The preprocessing steps included slice timing, spatial realignment, normalization to the Montreal Neurological Institute template (resolution of 3 mm × 3 mm × 3 mm) and spatial smoothing with a 6-mm full width at half maximum Gaussian kernel. It was planned that subjects whose translational head motion exceeded 2 mm or rotational motion exceeded 2° during scanning would be excluded, but none of the participants in the present study were excluded based on this criterion. Finally, we removed linear trends from time courses, performed temporal bandpass filtering (0.01–0.08 Hz) and regressed out the nuisance signals such as the six head-motion parameters, global mean, white matter signals, and cerebrospinal fluid signals.

### ALFF Analyses

The time courses were converted to a measure of frequency using a fast Fourier transform algorithm and the averaged square root of each spectrum across 0.01–0.08 Hz at each voxel was determined as the ALFF value. The framework for performing ALFF analyses can be seen in Figure S1 in Supplementary Material (an example of an ALFF analysis process in one voxel). The whole-brain voxel-wise ALFF was calculated in each participant in the patient group and healthy control group. Two-sample *t*-tests were performed to find areas that showed significant differences in ALFF between children with epilepsy, both before and after surgery, and the controls. The significance threshold of the two-sample *t*-test was set at *p* < 0.001 (*t* > 3.348) and a minimum cluster size of five voxels. The differences in ALFF between the pre- and postoperative scans in the patients group were also tested using paired samples *t*-tests. The significance threshold was set at *p* < 0.005 (*t* > 3.355) and a minimum cluster size of five voxels. We used this threshold in the paired samples *t*-tests considering a similar *t* value was used for both *t*-tests. Age and gender were controlled as covariates in all the above statistical analyses.

### Correlations between the ALFF and Clinical Characteristics in Patients

The regions that showed significant changes in ALFF following surgery were selected as regions of interest (ROIs) for *post hoc* analyses. Average ALFF values in each ROI were extracted. The percentage changes following surgery in ALFF in each ROI were calculated with the following formula: (post–pre)/pre × 100.

Partial correlation analyses were used to analyze the correlation between the percentage changes in ALFF values in each ROI and the age of epilepsy onset. The clinical factors, such as the interval between the two scans and the interval from epilepsy onset to surgery, were selected as control covariates. We used an uncorrected statistical significance level of *p* < 0.05, as these analyses were exploratory.

### FC Analyses

The filtered imaging data were then analyzed to remove the sources of spurious variance through linear regression, including the six parameters of head motion, the CSF signal, and the white matter signal. Time series of voxel within each ROI were averaged and corrected with the average time series of the other ROIs. Correlation values were then *z*-transformed to the mean of the whole sample. A two-sample *t*-test for statistical analysis of FC for each pair of ROIs was performed, comparing the patient and control groups both before and after treatment. To compare pre-surgical and post-surgical FC for each pair of ROIs in the patient group, we performed paired *t*-test analyses. We used an uncorrected statistical significance level of *p* < 0.05 as these analyses were exploratory.

## Results

### Between-Group ALFF Analyses

Before surgery, the patient group showed a higher ALFF in the bilateral thalamus, putamen, temporal lobe, and cerebellum compared with the control group. An increased ALFF was also observed in the left pallidum, the left precuneus, the right fusiform, and the right hippocampus relative to the controls. In contrast, a decreased ALFF was observed only in the right post-central gyrus.

**Figure 2:**
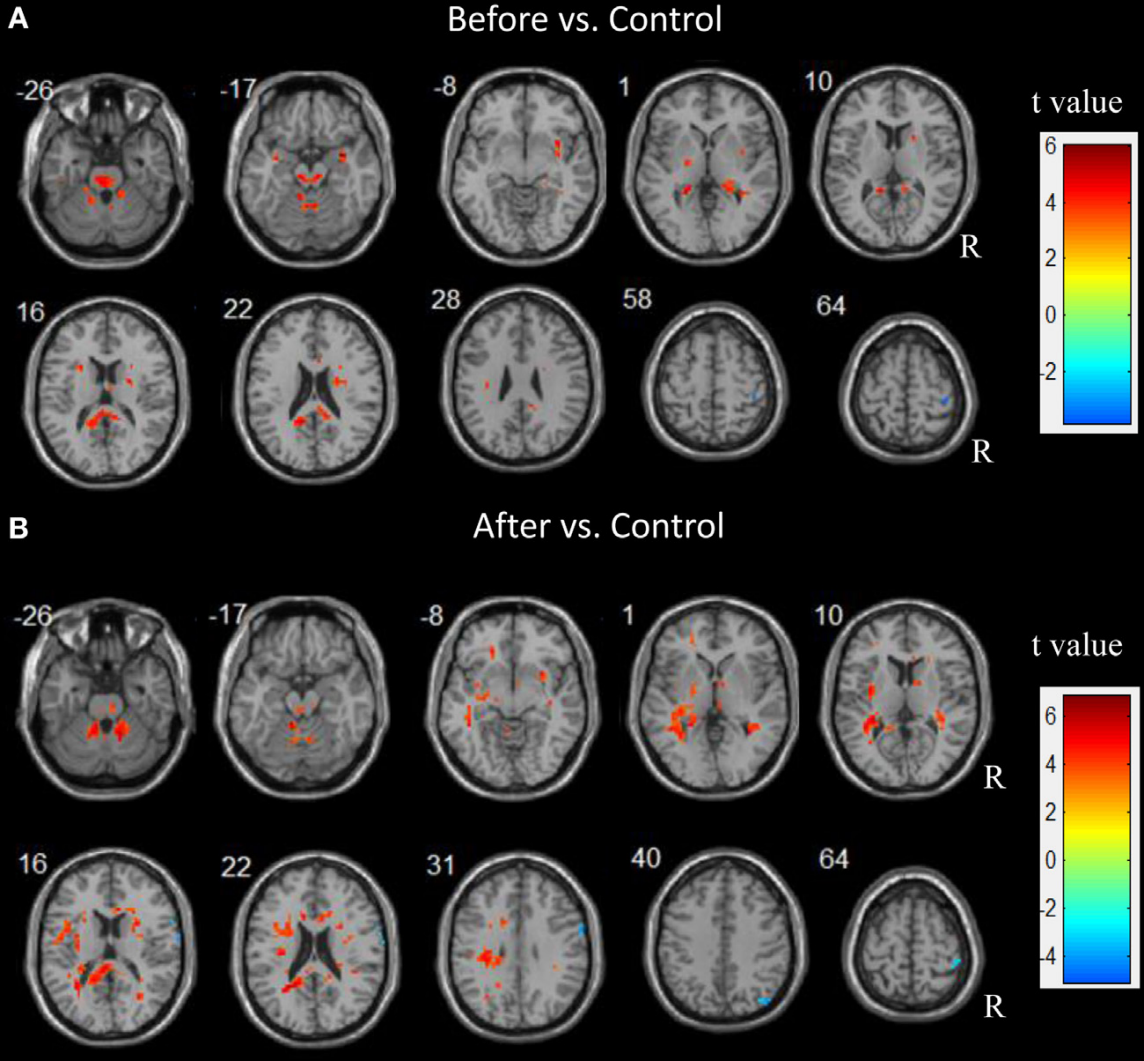
Abnormal spontaneous brain activity in children patients before and after epilepsy surgery. Increased amplitude of low frequency fluctuations (ALFF) are showed in hot color. In contrast, decreased ALFF are showed in cold color.

After surgery, the patients showed an increased ALFF in the left parahippocampus, precuneus, orbital middle frontal gyrus, inferior parietal lobule (IPL), and insula relative to the controls. Additionally, an increased ALFF was seen in the bilateral pallidum and the right thalamus, the putamen, the precentral gyrus, the orbital middle temporal gyrus, and the cerebellum. A decreased ALFF was observed in the right hemisphere including the angular, pre- and post-central gyri. Figure 2 depicts the significantly different ALFF values between the control and patient groups.

### Paired Samples *t*-Test Analyses in Patients

**Figure 3:**
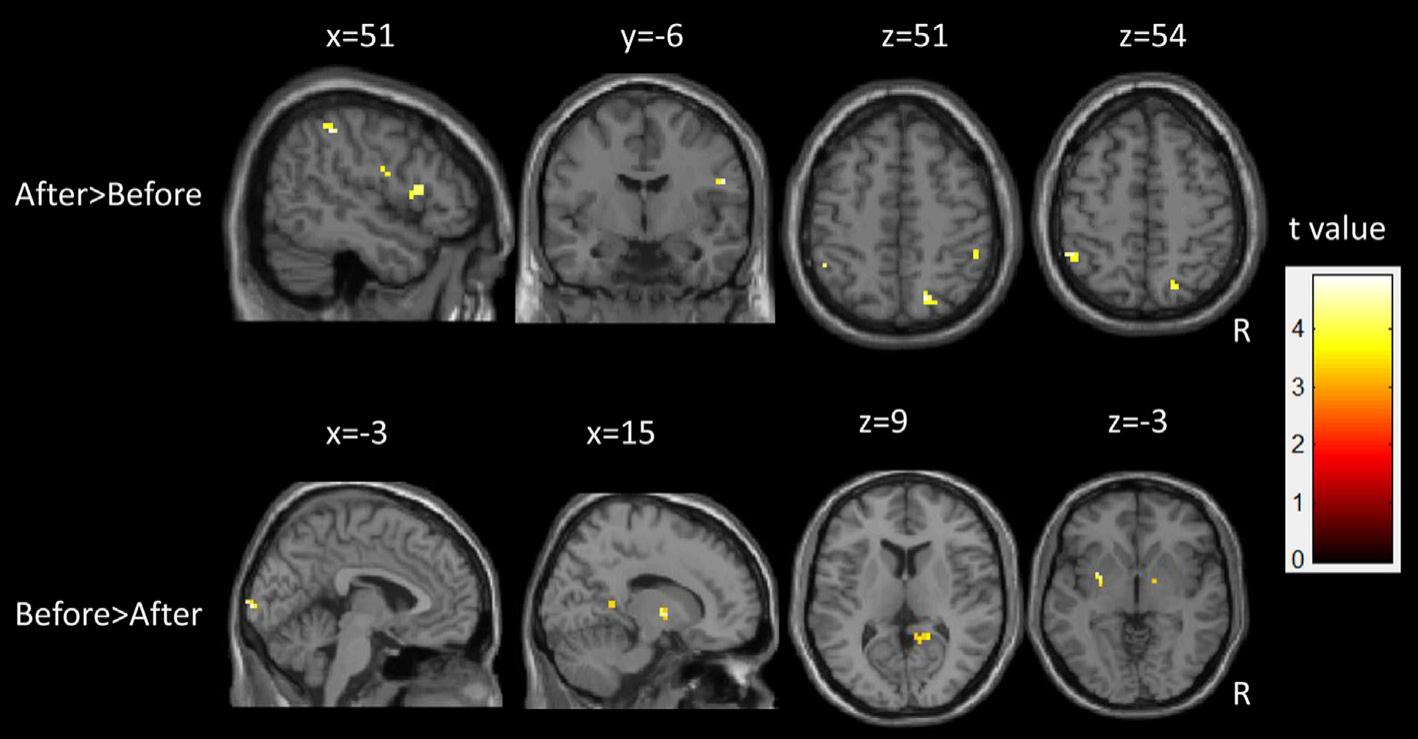
Longitudinal changes of spontaneous brain activity in children with medically refractory epilepsy from pre- to post-surgery.

Following epilepsy surgery, there were significant increases in ALFF in the bilateral IPL and right superior parietal lobule. The right post-central gyrus and inferior frontal gyrus (IFG) also showed significant increases in ALFF after epilepsy surgery. There were significant decreases in ALFF in the left putamen, the bilateral calcarine cortex, the right thalamus, and the middle cingulum after epilepsy surgery. There group differences are shown in Table 2 and Figure 3.

**Table 2:** Summary of significant activations between pre- and postoperative conditions in the patient group from the whole-brain analysis.

Comparisons	Statistical values			Coordinates anatomical location			
	Cluster size	*t*-Value	*p*-Value	*x*	*y*	*z*	Region
**After > before**							
	8	4.09	0.002	51	15	9	R Oper Inf frontal gyrus
	6	4.56	0.001	54	−6	24	R post-central gyrus
	6	4.92	0.001	51	−39	48	R Inf parietal lobule
	9	4.64	0.001	18	−69	51	R Sup parietal lobule
	8	4.53	0.001	−48	−48	54	L Inf parietal lobule
**Before > after**							
	5	5.43	0.000	−30	3	−3	L putamen
	7	5.57	0.000	15	−6	0	R thalamus
	5	6.01	0.000	−3	−102	3	L calcarine
	13	4.12	0.002	15	−46	6	R calcarine
	7	3.95	0.002	6	−18	30	R mid cingulum

*The MNI coordinates and t-values for the local maxima of the centers of the voxel clusters*.

*R, right hemisphere; L, left hemisphere; Inf, inferior; Oper, operior; Sup, superior; Mid, middle*.

*Threshold for significant clusters reported here was set at p < 0.005 and cluster size of 5*.

### Correlations between the ALFF and the Age of Epilepsy Onset

**Figure 4:**
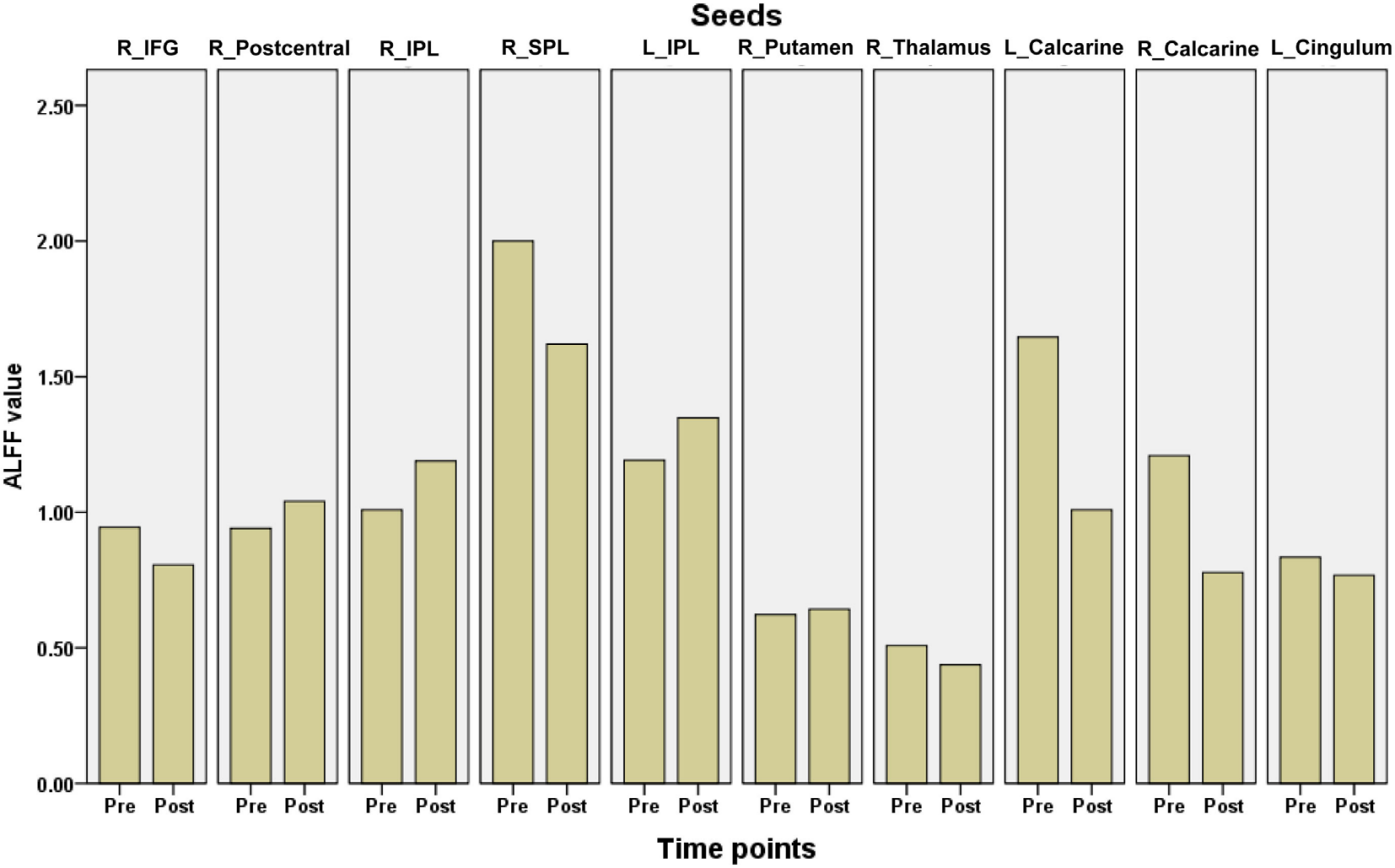
The mean amplitude of low frequency fluctuations (ALFF) values of each region of interest in children with medically refractory epilepsy from pre- to post-operation. pre, pre-surgery; post, post-surgery.

Ten clusters that showed significant changes in the ALFF following surgery were selected as ROIs for the correlation analyses. The mean ALFF values of each ROI were calculated as shown in Figure 4. The percent changes in ALFF and partial correlation analyses results are shown in Table 3. There was a significant positive correlation between the age of epilepsy onset and the percentage changes in ALFF values extracted from the left putamen (*r* = 0.808, *p* = 0.008), the left calcarine cortex (*r* = 0.812, *p* = 0.007), the right calcarine cortex (*r* = 0.625, *p* = 0.049), and the right middle cingulum (*r* = 0.894, *p* = 0.001).

**Table 3:** Partial correlation analyses between the percentage change of amplitude of low frequency fluctuations (ALFF) following surgery and age of epilepsy onset.

	Regions	ALFF values	
		*r*-Value	*p*-Value
Age of epilepsy onset	R Inf frontal gyrus	−0.257	0.270
	R post-central	−0.204	0.314
	R Inf parietal lobule	0.091	0.415
	R Sup parietal lobule	0.124	0.385
	L Inf parietal lobule	−0.221	0.300
	L putamen	0.808	**0.008**
	R thalamus	0.043	0.460
	L calcarine	0.812	**0.007**
	R calcarine	0.625	**0.049**
	R mid cingulum	0.894	**0.001**

*Results of partial correlation, control for the interval of two scannings, and the interval from epilepsy onset to surgery as covariates. Values in the table were Pearson’s coefficients (*r*) and the corresponding *p*-value (one-tailed). Significant correlation results were marked in bold*.

*Inf, inferior; Mid, middle; Sup, superior; R, right hemisphere; L, left hemisphere*.

### FC Analyses within the ROIs

**Figure 5:**
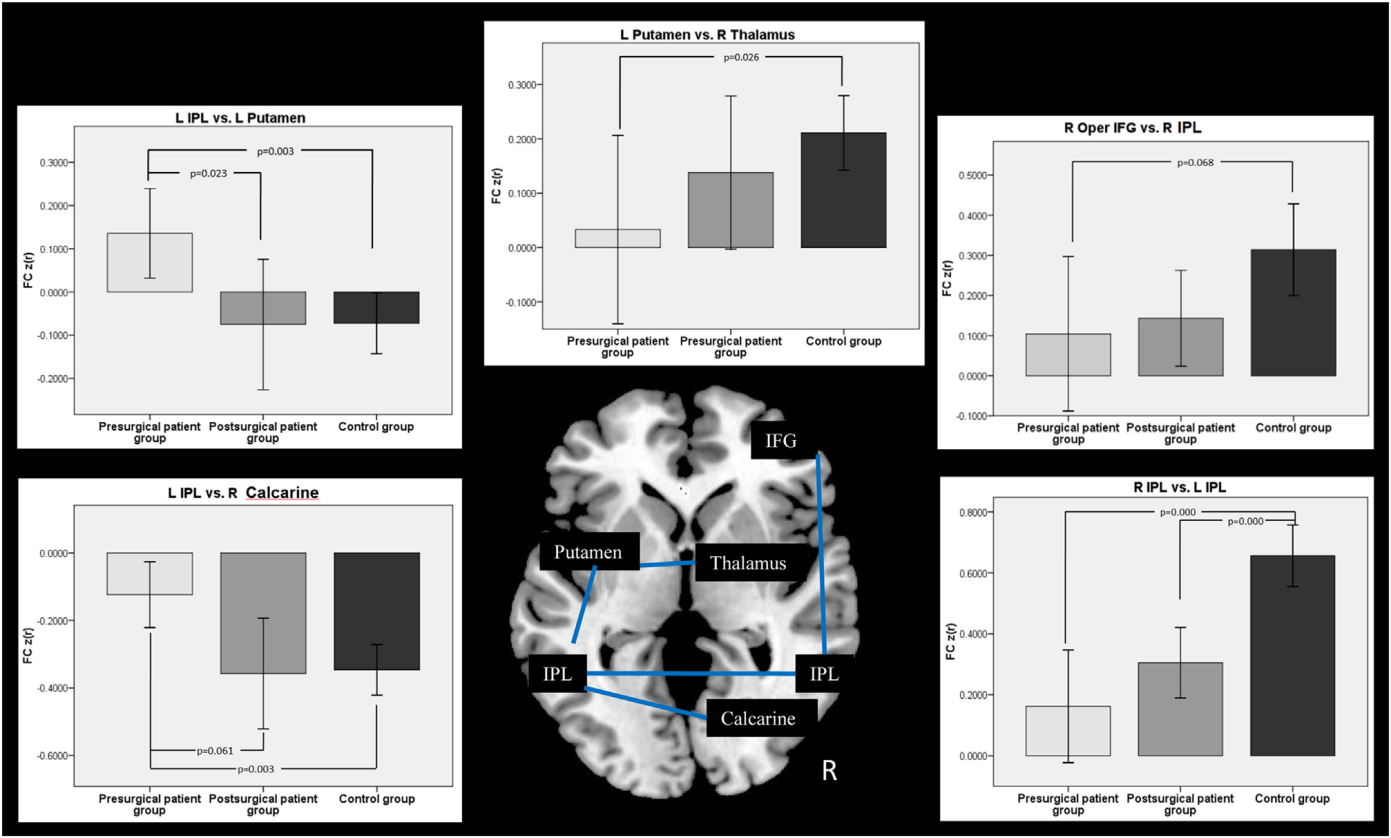
Comparison of functional connectivity (FC) among groups. Two-sample *t*-test was used between the patient and the control group, while two-pair *t*-test was used in the patient groups from pre- to post-surgery.

Connectivity between the time courses within 10 ROIs was calculated. The pairs of ROIs showed significant changes between groups were selected and displayed in Figure 5. FC analyses in patients showed that the connectivity between the left IPL and the left putamen was decreased nearly to the level of normal controls after the surgery. The connectivity between the left IPL and the right calcarine was disrupted in the patient group before surgery. After surgical intervention, this disrupted intrahemispheric connectivity was restored nearly to the level of that in the normal control group. The connectivity between the left putamen and the right thalamus was disrupted in the patient group at the pre-surgical scan compared with that of the controls. This disrupted connectivity was reorganized after surgery. We found that patients with MRE showed a significantly decreased bilateral IPL connectivity at both pre- and post-surgical scans compared with the controls. Compared with the controls, the FC between the right IFG and the right post-central gyrus showed a nearly significant decrease in the patient group before surgery. With the surgical intervention, this connectivity was increased but not reached the level of the control group.

## Discussion

This study demonstrates the functional consequences of surgery on brain functional networks in children with MRE. We used a longitudinal method with resting-state fMRI to explore aberrant intrinsic brain activity from pre- to post-surgery. We found that the ALFF values were increased significantly in the bilateral parietal lobe and right frontal lobe after surgery. In contrast, the ALFF values decreased significantly in the deep nuclei (such as the left putamen, bilateral calcarine, right thalamus, and middle cingulate gyrus) with surgical intervention. The percentage changes in ALFF values showed decreases in these regions and these values were correlated with the age of epilepsy onset. FC analyses further demonstrated a reorganization of FC architecture after surgery, especially the pathways of the left IPL vs. the left putamen and the left IPL vs. the right calcarine. These changes in spontaneous brain activity and FC architecture after surgical intervention are consistent with each other, which might indicate that the disrupted functional interactions were reorganized after surgery. The correlation between brain activity and clinical characters implied that the age of epilepsy onset might affect the brain functional organization. Overall, our results suggest that early and successful surgery in children with MRE may potentially induce brain functional plasticity. The age of epilepsy onset may affect children’s brain functional reorganization after surgery.

### Postoperative Spontaneous Brain Activity Changes as Measured by ALFF

Generalized epileptic discharges that are related to activation in the thalamus along with deactivation in the default mode areas have been reported in a large body of literature, suggesting seizure generation, and the suspension of brain default function (15, 17, 20). In the present study, resting-state fMRI data in children with epilepsy were collected and brain activity patterns were measured using ALFF values. The patient group showed an increased ALFF in the subcortical structures and decreased ALFF in the post-central gyrus before surgery. The results were consistent with previous studies and implied functional brain damage caused by epilepsy seizures. After surgery, the seizures were controlled and the abnormal brain activity in the patient group showed an adaptive process. These results might implicate that epilepsy surgery is an established treatment for the selected patients with MRE in the present study, which can induce brain reorganization. In the functional domain, this adaptive process after epilepsy surgery can re-establish the normal brain function that is disrupted by epilepsy seizure. For example, Wong et al. (9) offered the neuroimaging evidence that the cortical language network is reorganized after an anterior temporal lobectomy. In our study, the brain activity patterns and connectivity patterns in some regions were altered after surgery. The brain activity changes following surgery in the present study may suggest the importance of epilepsy surgery in cases of MRE in infants and young children (21, 22). In the present study, the ALFF values in the deep nuclei were decreased after surgery, which implied that epilepsy damage of the putamen and the thalamus were prevented and even showed reorganization after surgery.

The significant changes in ALFF values within these regions after surgery can be explained by their functional role. The significantly decreased ALFF values seen in the thalamus after surgery are consistent with the known important role of subcortical structures in the generalization of epileptic seizures (23). The thalamus is an essential node involved in epilepsy networks. This region plays an important role in the initiation, propagation, and inhibition of epileptic activity (15, 24). Generalized epilepsy seizures spread out through the cortical–subcortical epilepsy network. This induces electrical discharges in the regions in the epilepsy network. In the present study, significantly increased ALFF values in the temporal lobe, cerebellum, and subcortical nuclei before surgery demonstrated that these regions would be affect by the epilepsy seizure. With surgical intervention, longitudinal changes in ALFF in the right thalamus implied that surgery intervention might control seizure activity in children. The ALFF values were decreased significantly after surgery, as no seizures generalized through the thalamus.

In addition, an increase in ALFF after surgery was found in the bilateral IPL, the right superior parietal area, and the frontal area. A large body of imaging studies have well-addressed the abnormalities of the frontal–parietal network in epilepsy patients (17, 20, 25, 26). The damage due to chronic seizures was controlled with epilepsy surgery. Functional impairments were recovered leading to increased brain activity in the parietal and frontal areas. Brain functional plasticity theory can also be used to explain these results. Previous studies have shown that human brain networks are plastic and can reorganize in response to the changes in the external environment or internal milieu (27, 28). Resting-state networks were reorganized after brain epilepsy surgery in parallel with the recovery of brain function (9, 10, 29). Longitudinal analyses showed that the ALFF values in the frontal–parietal network were increased significantly from pre- to post-surgery in our study. These results demonstrate that the parietal and frontal areas were sculpted by epilepsy surgery.

### Correlations between the Percentage Changes in ALFF and Clinical Characteristics

The ALFF changes in fMRI signals have been suggested to be associated with clinical characteristics, such as the number of interictal epileptiform discharges or epilepsy duration (15, 17). Epilepsy duration was the most frequently used factor to calculate the neuroimaging prediction degree. In the present study, we used the patients’ age of epilepsy onset as a clinical factor to calculate the correlation with the neuroimaging results. We selected this clinical characteristic because our research subjects were children with MRE. Ongoing seizures would affect the normal developmental trajectory of these children’s brains. Our statistical results of the ALFF values between the pre-surgical group and the controls confirmed this expectation. The post-surgery seizure records indicate that all patients were seizure free after surgery. Once seizures are controlled after epilepsy surgery, the interrupted development in these children can potentially recover. Two prospective population-based observational studies have shown that early epilepsy surgery in infants and young children with MRE is important for improving brain activity (22, 30). A recent review in the domain of pediatric epilepsy also highlights the importance of early intervention of epilepsy surgery to brain recovery (21). Based on these previous studies, we speculated that children’s pre-surgical clinical condition might also have some predictive value regarding on brain reorganization after surgery.

Therefore, we selected children’s age of epilepsy onset as our focus to study whether it is possible to predict children’s functional brain status after surgery. As expected, our correlation results demonstrate that the children with a younger age of epilepsy onset showed a large decrease in ALFF values from pre- to post-surgery in the bilateral calcarine, left putamen, and right middle cingulum. In these analyses, the age, sex, interval between the two scans, and the interval from epilepsy onset to surgery were all controlled. The partial correlation results implied that the age of epilepsy onset in children with MRE can, to some extent, predict the degree of brain activity change after epilepsy surgery. A child with an earlier epilepsy onset combined with an early referral for epilepsy surgery evaluation can potentially have improved brain activity after surgery.

### Postoperative Brain Changes in FC

Functional correlations between the epilepsy-related areas in each group were calculated in the present study. Compared with the controls, the patients with MRE showed a reduced connectivity between the right superior IFG and the right IPL. This difference was no longer significant post-surgery, which may represent a normalization of this functional pathway following surgery. Previous resting-state FC studies in epilepsy have shown disconnected connectivity to nodes within the diseased hemisphere (13). Another study in patients with mesial temporal lobe epilepsy showed that FC decreases bilaterally (31). In addition, they found the number of decreased links was significantly higher in the epileptogenic side. In the present study, we flipped the epilepsy type to the right hemisphere. Therefore, we consider the diseased hemisphere in the present study to be the right hemisphere. Decreased FC within the right hemisphere was consistent with previous studies and provides evidence that functional interactions are dependent upon structural connectivity. Postoperatively, FC between the right IFG and the right IPL was partially increased, which demonstrated the reorganization of FC architecture with epilepsy surgery.

Furthermore, participants with epilepsy showed reduced connectivity between hemispheres, such as between the left IPL and the right calcarine, the left IPL and the right IPL, the left putamen and the right thalamus. The FC between the left IPL and the left putamen was increased significantly in patients before surgery. These results were consistent with the previous view of brain functional network changes after epilepsy or lesions (31–33). A previous study has shown that the interhemispheric FC in epilepsy patients was decreased compared with that in normal subjects (33). This study also showed that there was increased intrahemispheric FC within the healthy hemisphere in patients. Therefore, the decreased interhemispheric FC results found in the present study might imply that children’s brain functional integration between the hemispheres was damaged after epilepsy. In contrast, the increased intrahemispheric FC between the left IPL and left putamen indicated that the healthy hemisphere may be playing a compensatory role to support cognition (34). After surgery, these differences in interhemispheric FC were no longer significant except in the bilateral IPL. The increase in interhemispheric FC values after surgery might represent a reorganization of the functional pathway between the both hemispheres. The FC values between the left IPL and left putamen after surgery were decreased to a range similar to that of the controls. This may indicate that surgery can restore the functional pathways between the two hemispheres. The increased FC values between the functional cortexes within the diseased hemisphere after surgery would indicate brain functional reorganization. Therefore, compensation of the regions in the healthy hemisphere was no longer playing an important role after surgery. Taken together, these FC results suggest that surgery-induced brain functional recovery might be the main reason for the pathway reorganization between the hemispheres or in the functional areas within the seizure focus hemisphere.

### Limitations

Although brain activity differences were identified from pre- to post-operation, the interpretations of these results should be made cautiously. The sample size of epilepsy patients in the current study is small. Larger numbers of patients will be required to identify consistent changes associated with successful epilepsy surgery across participants. The selected patients in the present study might suitable for surgery to controlling the seizure. Some children patients with MRE might not achieve satisfy results after surgery. This type of patients should also be considered in the future study. Another limitation of the current study was the use of the brain reversal method. Although this method is usually used in brain lesion studies, this operation can also introduce certain deviations to the analyses. In future studies, larger numbers of patients will be needed to divide the patients into left- and right-side types of epileptic pathogency. Third, imaging data in the epilepsy children were only collected at two-time points: pre-surgery and once post-surgery (mean of 79 days later). A longitudinal study with more time points should be designed to describe the long-term effects of epilepsy surgery on children’s brain development. Finally, the intervals between the surgery and the second fMRI scan varied from 0.5 to 7 months. The sedatives were used in some patients during the scanning but others were not. Same imaging protocol was used for the pre- and post-surgical scans in all patients. We realized that all of these factors might affect our final results. Future studies should consider and control for these factors to improve the reliability of the results.

## Conclusion

The present study investigated epilepsy surgery-induced changes in brain activation and FC patterns in a group of 10 children with MRE using resting-state fMRI. Longitudinal analyses found that ALFF values in the parietal and frontal areas were increased significantly after surgery. In contrast, the ALFF values in the deep nuclei were decreased significantly after surgery. The percentage changes in ALFF values in the deep nuclei areas were correlated with the age of epilepsy onset. Here, FC analyses demonstrated a restoration of FC architecture after surgery, especially in the functional pathways between the both hemispheres or connecting the functional areas within the seizure focus hemisphere. These changes in brain activity and FC after surgical intervention might indicate that the disrupted functional interactions were reorganized after surgery. All these results provide preliminary evidence that the age of epilepsy onset might affect the brain functional organization. The current study can aid in understanding the neural mechanism underlying the functional plasticity following surgery in children with MRE.

## Ethics Statement

Written informed consent was obtained from all parents in accordance with the standards of the Declaration of Helsinki. The Ethics Committee of the Shenzhen Children Hospital approved this study.

## Author Contributions

YL, QC, KW, WH, and DA conceived and designed the experiments. ZT, QC, JW, FW, and YG performed the experiments. YL, KW, XM, and YW analyzed the data. YL, KW, and WH contributed reagents/materials/analysis tools. ZT, FW, QC, and DA responsible for patient management and conceptualized the study. YL, JW, and KW wrote and revised the paper.

## Conflict of Interest Statement

The authors declare that the research was conducted in the absence of any commercial or financial relationships that could be construed as a potential conflict of interest.

## Supplementary Material

Figure S1 Framework for performing amplitude of low frequency fluctuations (ALFF) analysis. The time courses of one voxel were converted to the frequency domain by using a fast Fourier transform. The averaged square root of the spectrum across 0.01–0.08 Hz at each voxel was taken as the ALFF value.
