# Supplementary figures and images for: Alterations in Spontaneous Brain Activity and Functional Network Reorganization following Surgery in Children with Medically Refractory Epilepsy: A Resting-State Functional Magnetic Resonance Imaging Study
Source: Front Neurol. 2017 Aug 3;8:374. doi: 10.3389/fneur.2017.00374 (PMC5541057; doi:10.3389/fneur.2017.00374)

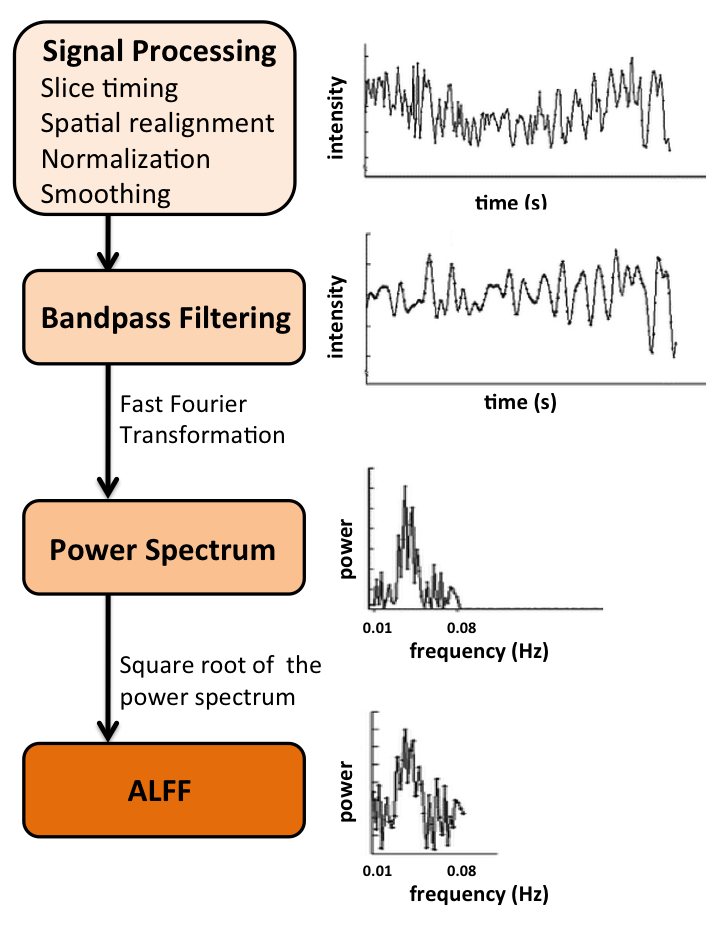

Supplement: Figure S1 — Framework for performing amplitude of low frequency fluctuations (ALFF) analysis. The time courses of one voxel were converted to the frequency domain by using a fast Fourier transform. The averaged square root of the spectrum across 0.01–0.08 Hz at each voxel was taken as the ALFF value. [file image_1.tif]
